# Navigating the Statistical Minefield of Model Selection and Clustering in Neuroscience

**DOI:** 10.1523/ENEURO.0066-22.2022

**Published:** 2022-07-07

**Authors:** Bálint Király, Balázs Hangya

**Affiliations:** 1 Lendület Laboratory of Systems Neuroscience, Institute of Experimental Medicine, H-1083, Budapest, Hungary; 2 Department of Biological Physics, Eötvös Loránd University, H-1083, Budapest, Hungary

**Keywords:** Bayes, bootstrap, clustering, cross-validation, information criterion, resampling

## Abstract

Model selection is often implicit: when performing an ANOVA, one assumes that the normal distribution is a good model of the data; fitting a tuning curve implies that an additive and a multiplicative scaler describes the behavior of the neuron; even calculating an average implicitly assumes that the data were sampled from a distribution that has a finite first statistical moment: the mean. Model selection may be explicit, when the aim is to test whether one model provides a better description of the data than a competing one. As a special case, clustering algorithms identify groups with similar properties within the data. They are widely used from spike sorting to cell type identification to gene expression analysis. We discuss model selection and clustering techniques from a statistician’s point of view, revealing the assumptions behind, and the logic that governs the various approaches. We also showcase important neuroscience applications and provide suggestions how neuroscientists could put model selection algorithms to best use as well as what mistakes should be avoided.

## Significance Statement

As neuroscience is becoming increasingly quantitative with “big data” approaches gaining a firm foothold in neurophysiology, neurogenetics and animal behavior, proper statistics for neuroscience is critically important. Nevertheless, probability theory and statistics is a dynamically evolving branch of mathematics; therefore, frequent cross-fertilization is required between probability theory and neuroscience. Statistical model selection, either implicit or explicit, is an integral part of data analysis in many neuroscience studies. Here, we review available and upcoming methods for statistical model selection, with an enhanced focus on cluster analysis as a special case and provide neuroscience examples for their application.

## Introduction

“All models are wrong, but some are useful (George Box, 1979).”

Broadly speaking, model selection encompasses all knowledge and assumptions about the underlying distributions for an observed sample. These assumptions have strong relevance to subsequent statistical decisions, like how to estimate central tendencies (e.g., mean, median), which statistical test to employ, how to describe and visualize the data. Arguably, the model selection trap most commonly walked into is performing it without noticing it. For instance, pitfalls of machine learning-inspired dimensionality reduction approaches have been demonstrated recently (Chari et al., 2021), suggesting that it may be useful to treat the choice among such algorithms as a model selection problem, asking which approach represents the important aspects of the data most faithfully (mine #1).

In simple cases, beaten paths are of help. For instance, limit theorems explain the laws that determine asymptotic distributions of the sums of independent random variables; as a result, normal distribution may be assumed for many types of data on the basis of the central limit theorem, or the Poisson process is a good model for “random” spiking of neurons, because of the Poisson limit theorem. Nevertheless, we suggest that conscious evaluation of such assumptions is not a futile exercise even in seemingly simple situations.

In less straightforward scenarios, one might have to resort to a statistical model selection approach, that is, to choose from possible models underlying a set of observations based on statistical principles (Konishi and Kitagawa, 2008). However, this is an inherently hard mathematical problem: the “true” model may or may not be among the investigated choices, and it is far from trivial to assess whether a model is better than a competing one. The probability of the observations is evaluated in face of a given underlying model, called the “likelihood,” providing a goodness-of-fit (GOF) measure with firm probability theory basis (for recent review on Bayesian model comparison, see Keysers et al., 2020). However, models of increasing complexity typically fit better (mine #2). This is easily illustrated by polynomial fitting: if we intend to fit n data points, a polynomial of degree *n*–1 (or higher) will provide a perfect fit. However, this is a poor argument to propose it as the “true” or even the “best” model. More likely, the data may be generated from a distribution with some level of stochasticity, better captured by a lower degree model.

Problems of this sort are frequently encountered in neuroscience. Generally, the GOF is discounted by measures of complexity; however, there is no single recipe for this equation, and as often happens, the multitude of proposed solutions demonstrates that none of them is perfect (Konishi and Kitagawa, 2008; James et al., 2013). While covering all important aspects of model selection would fill volumes, we focus on model selection problems especially relevant for neuroscience, emphasizing dos and don’ts, in the following (for the list of common “mines” of model selection discussed, see Table 1).

**Table 1:** List of common “mines” of model selection and clustering discussed in the paper

	Issue	Suggestion	Example
Mine #1	Selecting models without noticing it	Be aware of the assumptions behind analysis methods; treat the choice among different algorithms as a model selection problem	Chari et al. (2021)
Mine #2	Overfitting with overly complex models	Use statistical model selection tools which penalize too many parameters	Polynomial fitting
Mine #3	Selecting from a pool of poorly fitting models might lead to false confidence	Simulate data from each of the tested models multiple times and test whether the real data are sufficient to distinguish across the competing models	Figure 1*b*
Mine #4	Different information criteria might favor different models	Consider the strengths and limitations of the different approaches (Table 2); simulated data can be used to test which model selection method is the most reliable for the given problem	Figure 1*c* (AIC favors overfitting), *e* (BIC chooses an oversimplified model), *f*; Evans (2019)
Mine #5	Model selection might be sensitive to parameters ignored by the tested models	Avoid model classes that are too restrictive to account for data heterogeneity	Chandrasekaran et al. (2018)
Mine #6	Cross-validation techniques are prone to overfitting	A data splitting approach was proposed by Genkin and Engel in which optimal model complexity is determined by calculating KL divergence	Genkin and Engel (2020)
Mine #7	Agglomerative hierarchical clustering is sensitive to outliers	Consider divisive methods	Figure 2*c*; Varshavsky et al. (2008)
Mine #8	K-means clustering might converge to local minima	Repeat several times from different starting centroid locations	Figure 2*e*, right
Mine #9	Number of clusters not known	Use the elbow method, gap statistics, or model selection approaches	Figure 2*e*, left

## Model Selection Based on Akaike Information Criterion

Is it one bump or two bumps in my plot? More formally, does the mixture of two Gaussians provide a better model than a single one? Is the dependence between the measurements linear, logarithmic, exponential or best described by a quadratic equation? At this point, we hit a roadblock: how to arbitrate between models of disparate complexity?

The most often-used statistical tools for model selection in neuroscience are so-called information criteria, stemming from the maximum likelihood concept (Akaike, 1969, 1973; Banks and Joyner, 2017). Generalized from autoregressive (AR) models, Akaike introduced “an information criterion” (AIC; known today as Akaike information criterion) to compare statistical models of different complexity (Akaike, 1974). The AIC stands on solid statistical basis, rooted in the Kullback–Leibler divergence (KL) of information theory (Seghouane and Amari, 2007; Seghouane, 2010). The KL divergence quantifies the difference of the true distribution of the data compared with that derived from the tested model. In other words, it can be thought of as the “information loss” (in bits) or the “coding penalty” associated with the imperfect approximation of the true distribution. If we can measure an unbiased empirical distribution, like the frequency of heads and tails when tossing a coin, in the limit of infinite coin flipping, its KL divergence from the true distribution, 0.5 probability for each outcome for a fair coin, will tend to zero (Shlens, 2014; Cavanaugh and Neath, 2019). Formally, the KL divergence of the distribution *P* from the distribution (model) *Q* is defined by

$$D_{KL}(P\|Q)=\overset{\infty }{\underset{-\infty }{\int }}p\left(x\right)\log\;\frac{p\left(x\right)}{q\left(x\right)}dx.$$

Important to the derivation of AIC, the KL divergence can be decomposed into entropy (information content) of *P*, denoted by *H(P)* and cross-entropy of *P* and *Q*, denoted by *H(P,Q)*:

$$D_{KL}(P\|Q)=\overset{\infty }{\underset{-\infty }{\int }}p\left(x\right)\log\;p(x)dx-\overset{\infty }{\underset{-\infty }{\int }}p\left(x\right)\log\;q(x)dx=H\left(P,Q\right)-H(P).$$

The model that minimizes this quantity (minimal AIC estimator; MAICE) is accepted (Akaike, 1974, 1978). Thus, AIC relies on comparing competing models by the difference of their KL divergencies with respect to the “true model” (here denoted by *P*). It is easy to see that this optimization problem only depends on the cross-entropy term, as the entropy of *P* is cancelled in the difference. The cross-entropy is mathematically tractable and relatively easy to estimate (Rao and Nayak, 1985); however, the commonly used maximum likelihood estimation (MLE) is not unbiased in this case, hence it has to be corrected by subtracting the estimated bias. The core of deriving AIC is the bias estimation procedure by approximating the cross-entropy with its Taylor series to the quadratic term, relying also on the central limit theorem and the strong law of large numbers (Akaike, 1973; Cavanaugh, 1997; Konishi and Kitagawa, 2008). This results in the formal definition of AIC as follows:

AIC=−2 ln (L^) + 2k,

where $\hat{L}$ is the maximum likelihood of the model and *k* is the number of free parameters (Akaike, 1973, 1974, 1978).

**Figure 1.**
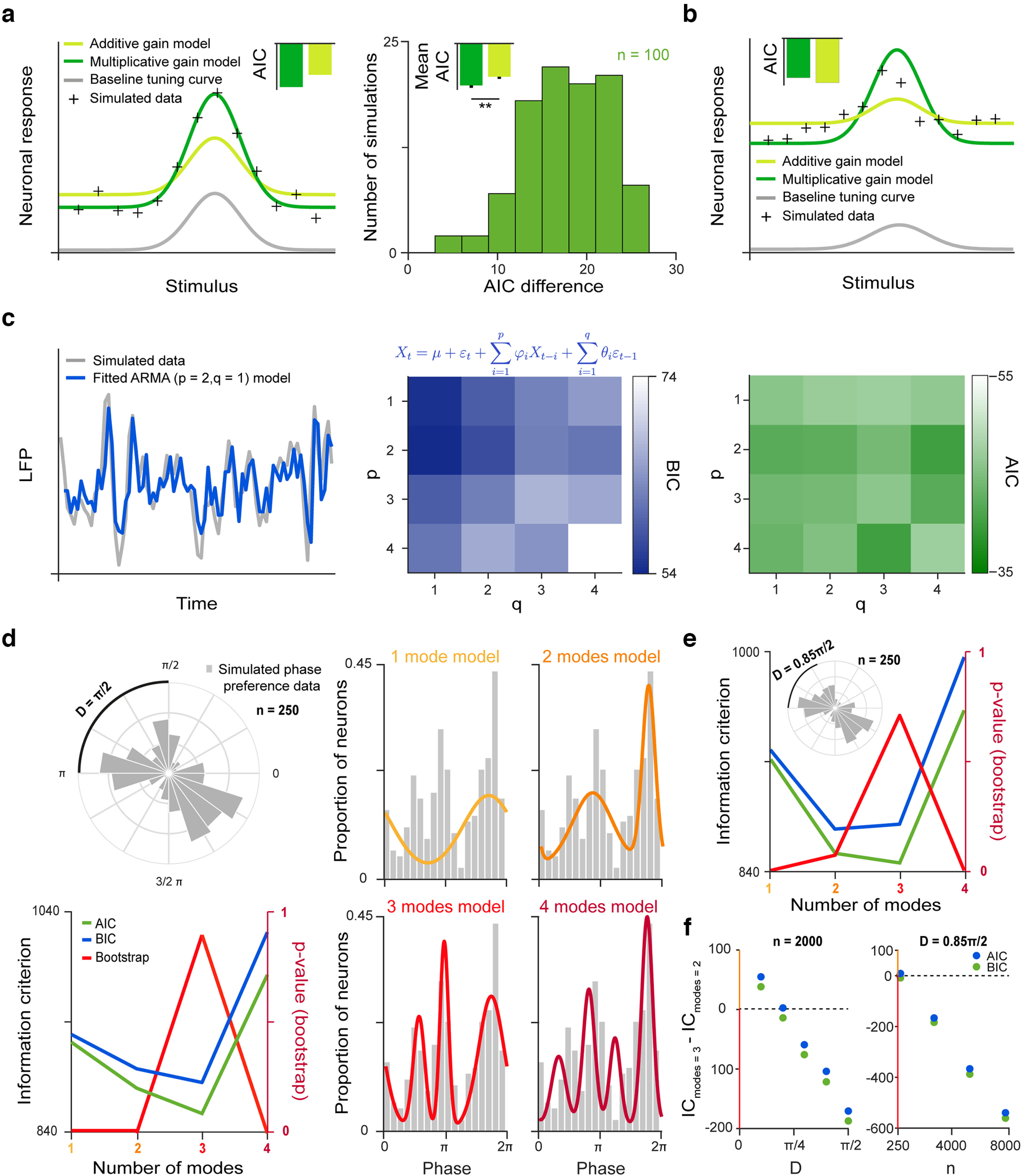
Examples of model selection problems in neuroscience. ***a***, Using MAICE to choose between competing models. Left, We used a bell curve to simulate neural responses as a function of stimulus features, generally referred to as a “tuning curve” (gray) and used a multiplicative gain model (*y = ax(s) + ε_i_*, where *y = x(s)* is the baseline tuning curve, *a* is a scalar and ε_i_ is a Gaussian noise term) to simulate a tuning curve change (black crosses). Next, an additive (light green) and a multiplicative model (dark green) was fitted on the simulated data. Smaller AIC value indicated that the multiplicative model fitted better (inset), as expected based on the simulation. Right, We performed *n* = 100 data simulations and calculated the difference between the AIC values of the competing fitted models. The histogram (and the mean values in the inset) demonstrates that in every case the multiplicative model outperformed the additive one. Error bars show standard deviation from the mean; **p < 0.01; two-sided bootstrap test. ***b***, A gain model with both additive and multiplicative components (*y = ax(s) + b + ε_i_*, where *y = x(s)* is the baseline tuning curve, *a* and *b* are scalars and *ε_i_* is a Gaussian noise term) was used to stimulate a tuning curve change (black crosses) relative to a baseline tuning curve (gray). Next, an additive (light green) and a multiplicative model (dark green) was fitted on the simulated data. While the best fit curves visible deviated from the simulated tuning curve, a smaller AIC value indicated that the additive model fitted somewhat better. ***c***, Using BIC to choose the best fitting ARMA model. Left, An ARMA (*p* = 2, q = 1) process was used to simulate LFP time series data (gray), where *p* denotes the order of the AR and q the order of the moving average component. Next, we fitted ARMA models on the data with different *p* and q values in the range 1–4. Blue trace shows the predicted data based on the best fitting *p* = 2, q = 1 model. Middle, BIC was calculated for each model, and the model with the lowest value (*p* = 2, q = 1) was chosen. Top, ARMA (p,q) model, where ϕ_i_ are AR parameters, θ_i_ are moving average parameters, ε_i_ are Gaussian noise terms, and μ is constant. Right, AIC was calculated for each model. AIC favored the more complex *p* = 2, q = 4 model over the expected *p* = 2, q = 1 model. ***d***, Demonstration of the use of information criteria and the parametric bootstrap technique for choosing the number of modes in a distribution. We simulated phase preference data of neuronal firing (*n* = 250) referenced to an LFP oscillation as the combination of three wrapped normal distributions (top left, D = π/2 refers to the phase difference between the mean of the two closest wrapped normal distributions). Next, mixture models of 1–4 von Mises distributions (circular analog of the normal distribution that closely approximates the wrapped normal distribution) was fitted on the distributions (right) using an expectation maximization algorithm for circular data (Czurkó et al., 2011). Minimal AIC and BIC, as well as maximal bootstrap *p* value correctly suggest that the model of three modes is the best fitting one. BIC is penalizing the higher mode models more than AIC (bottom left). ***e***, Same as panel ***d*** (left), but with D = 0.85 π/2 phase difference parameter. While minimal AIC and maximal bootstrap *p* value still suggest that the model of three modes is the best fitting one, BIC favored a simpler model with two modes. ***f***, Information criterion difference between the two-mode and three-mode models as a function of the phase difference parameter D (left, *n* = 2000) and the sample size (*n*, at D = 0.85 π/2). Color coding of the *y*-axis reflects the favored model (orange: 2 modes; red: 3 modes). If the modes are well-separated and the sample size is sufficient, both AIC and BIC choose the correct three-mode model, while they can fail for low sample size or less distinguishable modes. At parameters where the information criterion differences are closer to zero, BIC might favor the two-mode model while AIC might correctly identify three modes.

We demonstrate a use case of MAICE in Figure 1*a* by fitting different models to capture the essence of a tuning curve change. In sensory systems, neurons are often characterized by their tuning properties, which describe their responsiveness to external stimuli along different dimensions (Butts and Goldman, 2006). In the visual system, neurons of the primary visual cortex or the visual thalamus are investigated in terms of their orientation tuning, describing the angles of visual stimuli they prefer (Hubel and Wiesel, 1959; Atallah et al., 2012; Hillier et al., 2017). Auditory neurons can be categorized by their frequency-intensity tuning, revealing tonotopical organization of auditory cortices (Kilgard and Merzenich, 1998; Froemke et al., 2007; Hromádka et al., 2008). More broadly, “tuning” refers to the external or internal variables that drive neuronal firing, e.g., the tuning of hippocampal pyramidal neurons can be defined in terms of physical Euclidean coordinates (Muller et al., 1987; O’Keefe, 1993; Dupret et al., 2010) and the tuning within face patches of the inferotemporal cortex is defined by shape and appearance features of standardized images of faces as stimuli (Chang and Tsao, 2017). Tuning curves change during learning revealing rules of plasticity (Kilgard and Merzenich, 1998; Froemke et al., 2007), as a function of attentional modulation (Maunsell and Treue, 2006; Disney et al., 2007; Lee and Maunsell, 2009; Krueger and Disney, 2019) or in response to optogenetic modulation of different neuron types (Adesnik et al., 2012; Atallah et al., 2012; S.H. Lee et al., 2012; Pi et al., 2013; Wilson et al., 2012). Therefore, they have become important tools to help understand cortical information processing (Seriès et al., 2004; Butts and Goldman, 2006; Lee and Maunsell, 2009). Tuning curve changes are often captured by additive or multiplicative gain modulation models, in which selecting the best linear gain model occurs as a typical model selection problem (Atallah et al., 2012; Pi et al., 2013; Hangya et al., 2014). In our example, we simulated a gain change by multiplying a baseline tuning curve with a scalar factor. The MAICE method correctly indicated that the simulated data were better explained by a multiplicative gain model. Moreover, repeating the simulation a hundred times revealed statistical superiority of the correct model.

## Limitations of AIC

Although MAICE is a strong tool, some caution should be raised. First, when the true model is not among the tested ones, comparing poorly fitting models with AIC may lead to a false confidence in a model that is marginally better than some others. In Figure 1*b*, we simulated a tuning curve by combining an additive and a multiplicative gain; both the purely additive and purely multiplicative model showed poor fit, still, AIC chose a “winner” (mine #3). A related caveat is that AIC, in itself, does not provide error bars; in other words, one might not have enough data to draw conclusions on two similar models, despite AIC being nominally smaller for one of them. When this might be the case, it is recommended to statistically test whether the data are sufficient to distinguish across the competing models. This can be done by simulating data points from each of the tested models with a sample size of the original dataset multiple times, and evaluating whether the difference in AIC is consistent across those simulations (Fig. 1*a*).

Second, as noted above, the heart of the derivation of AIC is a correction that eliminates the bias introduced by the specific mode of MLE employed for the formula. However, this bias estimation only works asymptotically; it is therefore not recommended to use AIC on small sample sizes. A “corrected” AIC (AICc) was proposed for these cases; it is worth noting, however, that AICc relies on more specific assumptions on the distribution of the underlying data (Cavanaugh, 1997; Brewer et al., 2016).

Third, AIC is based on MLE, which is widely used and has strongly established, favorable statistical properties. Nevertheless, it may yield unstable parameter estimates for complex models, and therefore, a range of “regularized” (or “penalized”) MLE methods are available (Konishi and Kitagawa, 2008; Jang et al., 2016; Chamroukhi and Huynh, 2019). Since AIC formulation does not provide a straightforward way to incorporate these more robust MLE techniques, the generalized information criterion (GIC) has been introduced, which provides a recipe for constructing novel information criteria (Konishi and Kitagawa, 2008). However, it will take further theoretical work to determine how to derive suitable information criteria for neuroscience based on GIC.

## Bayesian Model Selection

The Bayesian approach to model selection is rooted in calculating the posterior probability of a candidate model, that is, how probable a given model is, provided the observed data (Kass and Raftery, 1995; Wasserman, 2000). Then, competing models are compared by their posterior probabilities, and the one that maximizes this quantity is selected.

In practical applications, the Bayes factor is often used, comparing the relative strength of evidence for two models *M_1_* and *M_2_*:

$$B=\frac{\text{Pr}(Data|M_{1})}{\text{Pr}(Data|M_{2})}.$$

Of note, the Bayes factor becomes the ratio of posterior probabilities of the models in case their prior probabilities are the same (the case of “uniform priors”), used for instance in hypothesis testing, where an equally plausible null and alternate hypothesis are compared (Keysers et al., 2020). Although the interpretation of actual Bayes factor values remains somewhat subjective, Jeffrey suggested in his classic work that a value over 10 should be regarded as “strong evidence” (Jeffrey, 1961), which notion is still generally accepted (Keysers et al., 2020).

In 1978, Schwarz introduced the Bayesian information criterion (BIC; also called Schwarz–Bayesian information criterion; Schwarz, 1978), formally defined by the following:

BIC=−2 ln (L^) + k ln (n),

where $\hat{L}$ is the maximum likelihood of the model (like in AIC), *n* is the sample size, and *k* is the number of free parameters. It has been shown that the BIC is an approximation of the logarithm of the Bayes factor; therefore, the BIC provides an easy-to-use tool for Bayesian model selection (Kass and Raftery, 1995).

To demonstrate a good use of BIC in a neuroscience context, we first go back to our example of polynomial fitting. MLE estimates trivially indicate better fits at higher degrees, because the class of n degree polynomials include all <n degree ones. As a counterbalance, information criteria penalize high number of parameters. AIC has a penalty of 2*k* (where *k* is the number of parameters), whereas BIC has a term ln(n)*k* (where n is the sample size), thus BIC implies a stronger penalty and hence tends to select simpler models (Brewer et al., 2016; Fig. 1*c–e*). Relatedly, AIC is often pictured as a method for selecting models for good “predictive accuracy” by penalizing sensitivity to spurious features of the data also known as overfitting, whereas BIC attempts to provide a good description of the fitted data as it penalizes for model complexity more broadly (Kass and Raftery, 1995; Evans, 2019; but also see Rouder and Morey, 2019). Mathematically, AIC is “asymptotically efficient,” minimizing prediction error as sample size tends to infinity, while BIC is “asymptotically consistent,” selecting the correct model, if it is in the tested pool, as sample size increases. They can also be seen as representing two different world views: in a complex world where the true generating model is unknown and may be unknowable, as suggested by the quote by Box we cited, one must resort to efficiency, where AIC wins. When one believes that a relatively simple model, included in the set of tested candidates, generates the data, BIC can pick the true model, while AIC does not come with such guarantees (Aho et al., 2014; Chandrasekaran et al., 2018).

A good use case of BIC is determining the optimal model order, for instance, when fitting AR models. The order of AR models determines the time scale at which previous information influences the “present” of a time series signal. In this case, competing models tend to be simple and we have an a priori bias toward smaller, more parsimonious models (Fig. 1*c*). Such analyses are typical in predictive time series analysis of EEG and fMRI traces (Muthuswamy and Thakor, 1998; Baajour et al., 2020). For instance, pathologic synchrony during epileptic activity can be detected and measured by analyzing the residual covariance matrix of an AR model fitted on multichannel scalp EEG recordings (Franaszczuk and Bergey, 1999). Similarly, cross-area interactions can also be quantified by multivariate AR models in fMRI recordings (Harrison et al., 2003; Stephan et al., 2004; Ting et al., 2015). Epileptic patients also show interictal spikes in EEG recordings, which can be efficiently detected using AR models estimated with a Kalman filter (Oikonomou et al., 2007). Figure 1*c* demonstrates the application of BIC to choose the best fitting AR-moving-average (ARMA) model for simulated local field potential (LFP) data. As indicated above, in such problems AIC may not pick the true model; indeed, in our example AIC favored a more complex model (mine #4; Fig. 1*c*, right).

## More Information Criteria

We may view the information criteria as methods for estimating the correct number of model parameters by finding their minimum. The BIC has the advantage over AIC that in the infinite limit of sample size, it yields a parameter estimate that converges to the true number of parameters with a probability of 1, called a “strongly consistent” estimate in statistics. Another strongly consistent information criterion was introduced by Hannan and Quinn (Hannan and Quinn, 1979), inheriting its favorable properties from the law of the iterated logarithm (Erdős, 1942):

HQC=−2 ln (L^) + 2k ln (ln(n)).

The penalty term grows very slowly as a function of sample size, which was suggested to lend the Hannan–Quinn Information Criterion better convergence properties compared with the BIC (Miche and Lendasse, 2009). Therefore, it is often used for determining the order of AR models (Sin and White, 1996; Miche and Lendasse, 2009), suggesting that it may have a yet unexploited place in the EEG and fMRI data analysis armament.

The deviance information criterion (DIC) is an extension of AIC, penalizing similarly for model parameters, but applying a different GOF measure, defined as the likelihood of the data averaged over the entire posterior distribution (Spiegelhalter et al., 2002; Celeux et al., 2006; Evans, 2019). By departing from the MLE-based GOF approach, it gained popularity in Bayesian model selection, when dealing with cases where maximum likelihood estimation is difficult (Celeux et al., 2006; Evans, 2019; Y. Li et al., 2020). While DIC assumes approximate normality of the posterior distribution (Spiegelhalter et al., 2014), Watanabe proposed a “widely applicable” (or Watanabe–Akaike) information criterion (WAIC) that does not rely on such assumptions (Watanabe, 2010a,b, 2013). Newest in this family, the “leave one out” information criterion (LOOIC) is similar to WAIC (Watanabe, 2010b; Gelman et al., 2014; Vehtari et al., 2017; Yong, 2018), but it has been proposed to yield more robust results in the finite case with weak priors or influential observations (Vehtari et al., 2017). Although these measures incorporate Bayesian notions, they can still be interpreted in terms of predictive accuracy, thus being advanced alternatives of AIC (Evans, 2019). Watanabe has made an attempt to generalize BIC as well, which resulted in the “widely applicable Bayesian” information criterion (WBIC) that seeks for the true model instead of minimizing predictive loss (Watanabe, 2013). Neural dynamics is usually best modelled by latent variable models, assuming a set of interacting hidden and observable variables (Sahani, 1999; Churchland et al., 2012; Vértes and Sahani, 2018; Pei et al., 2021), and doubly stochastic processes, where the dynamics is described by a random point process with varying intensity (Cunningham et al., 2008; Latimer et al., 2015). In these cases, one needs to apply the abovementioned information criteria relying on Bayesian GOF estimations (Latimer et al., 2015); therefore, we expect that these novel approaches will soon gain popularity in neuronal modeling and complex data analyses.

It is still debated among statisticians which information criterion is better and when (Konishi and Kitagawa, 2008; Maïnassara and Kokonendji, 2016). Since choosing the penalizing term will remain somewhat arbitrary, there likely will not be “one information criterion to rule them all.” Indeed, it is possible that different information criteria will favor different models, without a clear argument on which particular criterion suits the statistical problem at hand best (mine #4). For instance, Evans conducted a systematic comparison of a number of information criteria for a specific class of evidence accumulation models of decision-making processes and found that while model selection approaches typically agreed when effect sizes were moderate to large, they could diverge in their conclusions for small or nonexistent effects (Evans, 2019). He concluded that one should opt for “predictive accuracy” approaches like AIC when the primary goal is to avoid overfitting and thus select a model with strong predictive value, whereas BIC performs better if the goal is to provide the best account of the present data (Shmueli, 2010; Evans, 2019). Going one step further, one might adopt a simulation approach to test which model selection approach is the most reliable for the problem at hand, much the same as in the Evans study.

When complex systems, like those that determine the exact firing activity of neurons, are considered, it is unlikely that our models will capture all aspects of the true generating model. However, model selection approaches will always announce a winner, which raises a set of issues (Chandrasekaran et al., 2018). First, it is conceivable that all of the tested models fall far from the generating process, in which case model selection will yield a misleading conclusion about the data (mine #3). Second, model selection may be sensitive to parameters of the generating model not captured by the tested models. In such cases, model selection will suggest a model that is closer to the data in statistical or information theoretical terms, but not necessarily conceptually (mine #5). This is detailed in an elegant paper revealing model selection pitfalls when arbitrating between ramp-like and step-like changes in firing rates of single cortical neurons (Chandrasekaran et al., 2018). As a suggestion, one should take multiple close looks at the data, and avoid model classes that are too restrictive to account for data heterogeneity (Chandrasekaran et al., 2018; Genkin and Engel, 2020).

As a take-home message, information criteria are strong tools to contrast competing models, but researchers should always ask “Is my data sufficient and appropriate to discriminate these models?” (for details, see above, Limitations of AIC), and if the answer is yes, cautiously conclude that “this particular information criterion provides a statistical argument for model A describing the data better than model B.”

## Model Selection Using Resampling Techniques

Resampling techniques are strong tools of modern statistics with firm mathematical foundations while having minimal assumptions on the statistics of underlying data. On the flip side, they require substantial, sometimes prohibitively large, CPU-power.

In cross-validation methods, originating from machine learning, the data are split into a “training” and a “test” set. A model can be validated by fitting on the training set and obtaining GOF statistics on the test set. In neuroscience, the leave-one-out cross-validation algorithm is often applied: the model is fitted on *n*–1 data points, and tested on the remaining one, repeated for all data points as the “test set” (Kohavi, 1995; Browne, 2000; Hastie et al., 2009; J.R. Cohen et al., 2010). A generalization is the leave-p-out; however, more CPU-intensive with increasing p. To reduce CPU-load, the k-fold cross-validation may be applied: instead of testing all combinations of p-sized subsamples, the data are split into k groups to obtain the GOF distribution. Of note, the law of total probability, characterizing the relationship of conditional and marginal probabilities, reveals a deep link between cross-validation techniques and the Bayesian concept of likelihood. Specifically, decomposing the marginalized log likelihood function by the chain rule provides a formula equivalent to the sum of leave-p-out Bayesian cross-validation errors (Sadtler et al., 2014; Fong and Holmes, 2020).

It should nevertheless be noted that cross-validation techniques have some often overlooked, unfavorable statistical properties. Namely, they are prone to overfitting, giving undue credit to more complex models (Gronau and Wagenmakers, 2019). Indeed, when complex, flexible models are applied to broadly capture data heterogeneity, cross-validation techniques at realistic data amounts may not be able to prevent overfitting (mine #6). To overcome this, a novel data splitting approach was proposed recently, in which the data are divided into two halves, and the optimal model complexity is determined by calculating KL divergence between the distributions corresponding to models of the same complexity fitted on the two datasets (Genkin and Engel, 2020). A caveat of this proposal is that the KL divergence rises more or less monotonically with model complexity; thus, an empirical threshold is suggested to determine the “optimal point” the robustness of which has to be determined by future studies.

Neuroscience applications include a wide range of classification problems, from linking fMRI or fNIRS data to human behavior (J.R. Cohen et al., 2010; Jiang et al., 2012) to categorizing stimulus responses of cortical neurons (de Vries et al., 2020). Recently, cross-validation techniques have gained additional momentum as machine learning techniques revitalize many areas of neuroscience (Savage, 2019; Yang and Wang, 2020), since they are considered the first choice model selection tools when fitting artificial intelligence (AI) models. In this regard, it is important to highlight the attempts to automate classification of neurons based on their morphologic (m-types), electrophysiological (e-types) and transcriptomic (t-types) characteristics (Armañanzas and Ascoli, 2015; Saunders et al., 2018; Gouwens et al., 2019, 2020; Que et al., 2021).

Of the resampling approaches, parametric bootstrap is a particularly useful technique, often overlooked in neuroscience. When testing models that can be described with a relatively small number of parameters (e.g., a mixture of Gaussians), one can generate a bootstrap set of simulated data from these models, and use a GOF measure to describe the relationship of the model and the simulated data. A bootstrap distribution of such GOFs can then be used to estimate the probability of the original data violating the tested model (Fisher, 1993; Czurkó et al., 2011). Thus, unlike information criteria only providing a relative score, one obtains a *p* value. The model with the highest *p* value (least rejected model) wins. A practical application of parametric bootstrap is arbitrating the number of modes in an empirical distribution, using a mixture of unimodal distributions described by few parameters (e.g., Gaussians). For instance, hippocampal neurons are often characterized by the systematic relationship between their action potentials and the dominant ongoing local population activity, the theta rhythm (Buzsáki, 2002; Klausberger and Somogyi, 2008; Czurkó et al., 2011; Buzsáki and Moser, 2013). Neurons are active at multiple phases of this oscillation (Klausberger and Somogyi, 2008; Czurkó et al., 2011), but is the observed circular phase histogram of hippocampal activity truly multimodal? We demonstrate the power of parametric bootstrap approaches on this example in Figure 1*d*. Of note, while AIC and BIC work well for sufficient sample sizes and well-separated modes, they can both fail for low sample sizes or if the modes of the generating model are less distinguishable (Fig. 1*e*,*f*).

Bootstrap techniques also differ from information criteria in their capabilities to evaluate standalone models, while AIC, BIC, and related methods can only perform comparison of competing models. As a middle ground, one may ask the question whether a given model is better than a minimalistic model that still captures selected features of the data. Bayesian decoding can be used to extract relevant features of the data in a model-free paradigm (Koyama et al., 2010; Kloosterman et al., 2014). In neuroscience, this approach may relate neuronal activity to external variables as a generalization of the concept of tuning (see above; Okun et al., 2012; Kloosterman et al., 2014), or aim at understanding interdependences within populations of neurons (Okun et al., 2012; Bányai et al., 2019). Maximum entropy models (Tkačik et al., 2013) are powerful tools to generate “minimal” models with appropriate constraints, reviewed elsewhere (Savin and Tkačik, 2017). We provide an overview of the model selection techniques most commonly used in neuroscience in Table 2.

**Table 2:** Advantages and limitations of model selection and clustering algorithms

Method	Advantages	Limitations	Suggestions
Statistical model selection			
Akaike information criterion (AIC)	Strong mathematical basis (KL-divergence)	May lead to false confidence in marginally better models	If critical, perform simulations to ascertain true differences among the tested models
	Easy to calculate	Difficult to test whether differences are significant	
	Suitable for comparing models of different complexity	Not suitable for low sample sizes	Consider AICc if its assumptions are met
Bayesian information criterion (BIC)	Strong mathematical basis (Bayesian statistics)	Asymptotic properties may not hold for complex (multi-parameter) models	BIC is more recommended for simpler models, especially when overfitting is a concern, e.g., deciding the order of an AR process
	Easy to calculate	Difficult to test whether differences are significant	Consider simulations, as for AIC
Resampling methods	No assumptions on data distributions	CPU-intensive	Parametric bootstrap and cross-validation are often the best choice for testing models with few parameters
	Provides a *p* value for each tested model	Does not always converge to the true model (statistically inconsistent in the M-closed case)	
Clustering			
Hierarchical clustering, agglomerative	Simple	CPU-intensive for large datasets	With careful consideration of choosing similarity measure, clustering rule and other parameters, the flexibility of hierarchical clustering can be used to its advantage; test the robustness of the results by exploring the parameter space
	Easy to interpret	Sensitive to outliers and choices of algorithms and parameters	
Hierarchical clustering, divisive	Includes more robust and CPU-efficient options	Sensitive to choices of algorithms and parameters	
K-means clustering	CPU-efficient	Requires a priori estimate of number of clusters	Ideal choice if number of expected clusters is known; explore robustness of results by starting the algorithm from different sets of centroids
	Does not rely on many parameters	May converge to local minima and not find the global optimum	

The last column provides suggestions on how to use.

## Clustering Problems

Clustering problems form a special class of model selection that deserves attention because of its broad usefulness in neuroscience. Neuroscientists often aim to identify groups with similar properties: action potentials that likely belong to the same neuron based on similar spike shape (spike sorting; Quiroga, 2012; Yger et al., 2018; Magland et al., 2020), neurons that likely belong to the same cell type based on similar gene expression profiles (Saunders et al., 2018; Gouwens et al., 2020) and cells that represent the same behavioral variable based on similar response patterns in task-performing animals (J.Y. Cohen et al., 2012; Hangya et al., 2014).

**Figure 2.**
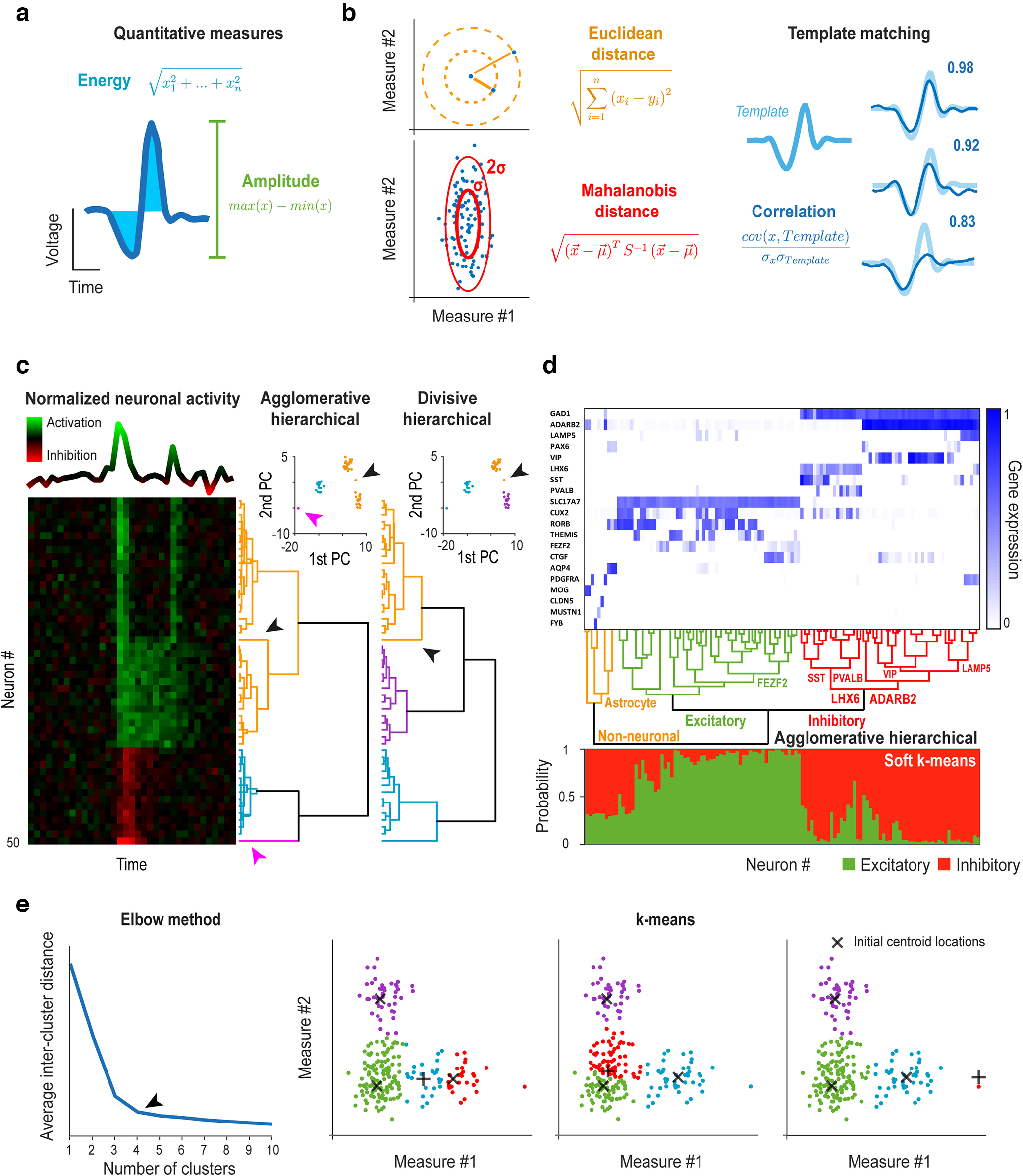
Examples of clustering problems in neuroscience. ***a***, Examples of simplified quantitative measures of waveforms often used in spike sorting. ***b***, Examples of distance measures used for quantifying similarity between data points and clusters often used in spike sorting. The Mahalanobis distance normalizes the standard deviation across dimensions, while template matching approaches are based on waveform correlations with predefined waveform templates. ***c***, Hierarchical clustering of simulated neuronal activity (peri-event time histograms) of *n* = 50 neurons with 3 (seemingly) very well separated groups (left). First, principal component analysis (PCA) was used to reduce dimensionality of the time series data. Second, agglomerative and divisive hierarchical clustering was performed in the space spanned by the first two principal components (right). Agglomerative clustering separated a single outlier cell (magenta arrow) in an earlier step (second) than the three main clusters, in contrast with divisive clustering. The two methods also differed in the clustering of a cell similar to more than one main groups (black arrow). ***d***, Clustering of human cells from multiple cortical areas based on RNA-sequencing data (*n* = 50,281 genes, publicly available at https://portal.brain-map.org/atlases-and-data/rnaseq/human-multiple-cortical-areas-smart-seq). Top, Trimmed mean expression of *n* = 20 marker genes. Middle, Agglomerative hierarchical clustering was performed based on the first 20 principal components, revealing the hierarchy of cell types (branches of the dendrogram were identified based on marker gene expression). Bottom, Soft K-means (k = 2) clustering was performed to assign probabilities to all cells of belonging to each of two main cell types, identified as excitatory and inhibitory based on marker gene expression. ***e***, Spike sorting of simulated action potentials (*n* = 221) using K-means clustering. Left, We applied the elbow method on the average intercluster squared Euclidean distance of all points to find the optimal number of clusters (k = 4, black arrow), based on 100 repetitions of K-means clustering for each k in the range of 1–10. Right, K-means clustering (k = 4) was performed with three different initial centroid locations (black crosses, 3 of which were kept at fix positions while one was changed), leading to surprisingly different clusters.

Clustering problems typically require solving a series of model selection problems (Konishi and Kitagawa, 2008; James et al., 2013). First, multidimensional data should be modeled by simplified quantitative measures that capture important variance from the neuroscience point of view, like action potential amplitude for spike sorting (Schmitzer-Torbert et al., 2005; Fig. 2*a*). Second, “similarity” of data points needs to be defined by an appropriate distance measure, most often the Euclidean distance between points in the space defined by this model (Schmitzer-Torbert et al., 2005; Fig. 2*b*, left). However, this may not be straightforward, for example in time series analysis dealing with time-resolved membrane potential, spike trains, LFP, EEG, ECoG, or fMRI, where linear or information theory-based correlation measures (Fig. 2*b*, right), potentially combined with dimensionality reduction techniques like principal component analysis, may be considered (Fig. 2*c*,*d*; Goutte et al., 1999; J.Y. Cohen et al., 2012; Thirion et al., 2014). Third, alternatives to “hard/complete” clustering, allowing cluster overlaps or probabilistic cluster assignments may be contemplated (Fig. 2*d*; Yang, 1993; Goutte et al., 1999).

There are two fundamental approaches to create the clusters (James et al., 2013). In hierarchical clustering, clusters are defined by merging data-points bottom-up in agglomerative, or splitting groups top-down in divisive clustering, either way creating a dendrogram of clusters (Fig. 2*c*). Agglomerative clustering is a popular choice, owing to its simplicity and ease of interpretation (Ward, 1963). However, agglomerative techniques are computationally heavy for large datasets, and particularly sensitive to outliers, since local properties determine their amalgamation rules (mine #7). These pitfalls can be overcome by special divisive methods that reduce such noise sensitivity by taking the global data distribution into account and have the computationally efficient option to stop once the required number of clusters is reached (Varshavsky et al., 2008). We demonstrate this on a simulated example of hierarchical clustering of peri-event time histograms of averaged neuronal responses (J.Y. Cohen et al., 2012; Hangya et al., 2015; Sadacca et al., 2016; Takahashi et al., 2016). We represented the peri-event time histograms by their first and second principal components to reduce data dimensionality and compared the results of agglomerative and divisive hierarchical clustering in Figure 2*c*. Another neuroscience application of hierarchical clustering considers groups or types of neurons based on transcriptomic information (C.L. Li et al., 2016; Faure et al., 2020). We demonstrate agglomerative hierarchical clustering of human cortical cells based on publicly available RNA-sequencing data (https://portal.brain-map.org/atlases-and-data/rnaseq/human-multiple-cortical-areas-smart-seq) in Figure 2*d*. In course of analyzing single cell transcriptomic data, the recently developed t-distributed stochastic neighbor embedding (t-SNE; van der Maaten and Hinton, 2008; Kobak and Berens, 2019) is often applied for dimensionality reduction (Harris et al., 2018; Saunders et al., 2018; Kobak and Berens, 2019). Although t-SNE faithfully reflects local structure, i.e., within-cluster distances, it does not preserve global structure (between-cluster distances), which renders it suboptimal for clustering. In 2018, McInnes proposed a novel dimension reduction algorithm he coined uniform manifold approximation and projection (UMAP), which, by a better choice for the cost function, based on KL divergence in t-SNE and cross-entropy in UMAP, results in better preservation of global data structure (McInnes et al., 2018). Therefore, while both t-SNE and UMAP are excellent visualization tools for high-dimension datasets, UMAP is more recommended for subsequent cluster analysis (McInnes et al., 2018; Diaz-Papkovich et al., 2021). The advantage of using nonlinear dimensionality reduction algorithms like UMAP for solving neuroscience problems is demonstrated by a novel UMAP-based spike sorting approach that can successfully sort cerebellar Purkinje cell recordings, a notoriously hard problem due the high degree of variability of simple and complex Purkinje cell spikes (Sedaghat-Nejad et al., 2021).

In contrast to hierarchical algorithms, a number of arbitrary cluster centers are assigned in K-means clustering, updated repeatedly based on proximity of data points to these centroids (Hastie et al., 2009). With a priori information on the number of groups and well-separated clusters, K-means is fast, efficient, and does not rely on a large number of potentially ambiguous choices. Nevertheless, these assumptions often remain unmet, when the flexibility of hierarchical clustering (choice of similarity measure and clustering rules), used wisely and cautiously, may provide better results. Furthermore, hierarchical clustering is deterministic, unlike K-means, which depends on the initial choice of centroids and might converge to local minima that can give rise to incorrect interpretations (mine #8). To avoid this, it is recommended to repeat K-means clustering several times using different initial centroid positions. We showcase K-means clustering on a spike sorting example (Fig. 2*e*). We simulated action potentials and sorted them into clusters corresponding to putative single neurons, mimicking a typical spike sorting problem in extracellular electrophysiology (Schmitzer-Torbert et al., 2005; Quiroga, 2012). Starting the algorithm from different centroid locations shows the sensitivity of K-means clustering to initialization (mine #8).

More robust results may be achieved by a relatively new technique called “spectral clustering” that combines K-means clustering with dimensionality reduction, however, at the price of losing the appealing simplicity of K-means clustering lauded above (Shi and Malik, 2000; Hirokawa et al., 2019). Additionally, fitting a mixture of Gaussians by expectation maximization (Jung et al., 2014) or other algorithms (Kántor et al., 2015) can be considered as an alternative to K-means clustering that allows operating with likelihood by proposing a statistical model (Fig. 1*d*).

An interesting algorithm based on physical properties of an inhomogeneous ferromagnetic model, called superparamagnetic clustering gained considerable popularity in neuroscience owing to its unsupervised nature that does not pose assumptions on the underlying data (Blatt et al., 1996; Domany, 1999). It has been used in a wide range of applications from spike sorting combined with wavelet spectral decomposition (Quiroga et al., 2004; Townsend et al., 2011) to morphologic classification of neurons (Zawadzki et al., 2010) to analyzing visual stimulus processing (Jasa et al., 2008).

In graph-like data structures where data points (“nodes”) are connected with links (“edges”), graph theory-based methods can be applied to detect clusters (“communities”) of the network. In such methods, a “modularity” measure is optimized that compares the link density inside versus outside the communities (Blondel et al., 2008). Lee and colleagues applied the graph-based Louvain community detection on spike waveforms of the macaque premotor cortex after nonlinear UMAP embedding (see above) and demonstrated the usefulness of this approach in revealing functional cell type diversities (Blondel et al., 2008; E.K. Lee et al., 2021). Of note, while the Louvain approach was developed to deal with extremely large graphs in a computationally efficient manner, its two-phase algorithm of finding high modularity partitions leaves the question open whether the order of considering the nodes throughout the algorithm can have a substantial effect on the results (Blondel et al., 2008). Nevertheless, Lee et al., showed that their approach resulted in stable clusters and outperformed Gaussian mixture model clustering applied on specific waveform features (E.K. Lee et al., 2021).

There is an important model selection problem often at the heart of clustering: how many clusters are there (mine #9)? A number of tools have been developed to aid this decision. The ratio of the between-cluster variance to the total variance monotonically increases as a function of the number of clusters, but typically flattens significantly at a point, called the “elbow” (Fig. 2*e*). The location of this bend is generally considered as an indicator of the appropriate number of clusters. A statistical approach to formalize this heuristic is the gap statistic (Tibshirani et al., 2001), based on comparing the total within-cluster variation with its expected value under the null hypothesis of no clusters present in the data. The optimal number of clusters is the one that maximizes this difference (the “gap”; Tibshirani et al., 2001). The gap statistics has been employed in spike sorting (Nguyen et al., 2014) and other clustering problems in neuroscience (Ito et al., 2018; Gwo et al., 2019), including fMRI-based connectivity analyses (Hahamy et al., 2015). As an alternative, most model selection approaches discussed above, including information criteria and parametric bootstrap for different number of clusters as competing models may be recruited for clustering problems.

## Conclusion

We showcased widely used model selection and clustering approaches especially relevant to neuroscience problems, also pointing to promising “up-and-coming” methods. Nevertheless, an exhaustive overview would stretch beyond the limits of this review. Most importantly, we would like to stress that model selection is a scientific field on its own right and urge neuroscientists to take conscious decisions about selecting the appropriate techniques and parameters, very much the same way as deciding on experimental design. The bad news is there is no free lunch or rules of thumbs that solve it all; however, the overwhelming good news is that a whole, exciting, and dynamically evolving world waits out there to be discovered and used to the full benefit of neuroscience.

### Data availability statement

No original data were generated.

### Code availability statement

We generated code in MATLAB 2016b (MathWorks) for the simulations presented in the figures, available at https://github.com/kiralyb/model_selection_mines.

## Synthesis

Reviewing Editor: William Stacey, University of Michigan Decisions are customarily a result of the Reviewing Editor and the peer reviewers coming together and discussing their recommendations until a consensus is reached. When revisions are invited, a fact-based synthesis statement explaining their decision and outlining what is needed to prepare a revision will be listed below. The following reviewer(s) agreed to reveal their identity: Thomas Kreuz.

The reviewers and I have discussed the article and find it to be interesting and important, but have identified several areas that must be improved.

1. The article currently suffers somewhat from an inconsistent target audience. We felt it lies somewhere between a primer for new students and a state-of-the-art description for experts. It also is not clear whether this is directed towards neuroscientists learning more about the models, or statisticians learning more about neuroscience. To start, we suggest assuming a target audience of neuroscientists, which is the readership of eNeuro. Then improve that particular focus. Currently, most concerns raised are what you would find in any regular stats textbooks, e.g. small sample sizes. This is predictable and does not push the field forward. On the other hand, we felt the introductions and long lists of citations and examples were distracting. Rather than an exhaustive review of examples with many details, this work should focus on the “statistical minefields” and how to avoid them. i.e. more of a statistical primer for neuroscientists, than a neuroscience review for statisticians. Doing so will improve the focus and allow better rigor.

2. The paper currently does not demonstrate or solve any “minefields” in model selection. All the simulated examples seem to be straightforward and there is no issue there in terms of selecting the right model. This is in stark contrast to recent papers that show specific issues with model selection and cross validation. The title lures the reader into thinking there are minefields with problems if the wrong model is chosen, but instead the paper itself seems to show that the chosen models always work, which was confusing to the reviewers. With the current title, we would expect some concrete examples of problems and ways to mitigate them. Here are three examples that do so:

a. Chandrasekaran C, Soldado-Magraner J, Peixoto D, Newsome WT, Shenoy KV, Sahani M. Brittleness in model selection analysis of single neuron firing rates. BioRxiv. 2018 Jan 1:430710. Demonstrates that model selection outcomes are dependent on the precise mathematical details of the formulation and if data deviate from the models tested then you get into all sorts of trouble. In particular, the figure 8 in that paper describes the various issues that can happen with model selection.

b. Genkin M, Engel TA. Moving beyond generalization to accurate interpretation of flexible models. Nature Machine Intelligence. 2020 Nov;2(11):674-83. Suggests a data splitting approach and looking for consistency across datasets is the way to find interpretable models.

c. Chari et al., The Specious Art of Single Cell Genomics. Shows how dimensionality reduction applied willy nilly to a 2D space will lead to distortions of the original space, which is essentially a problem of model selection of restricting data to 2 dimensions.

3. Related to #2, perhaps you could provide an example where the true model is not in the classes that were tested and show that model selection provides different answers based on AIC or BIC. We do not demand new experiments/simulations for the resubmission, but include such an example (even one from other publications) would make this point much more rigorously. Alternatively, if you had unpacked the KL divergence with excellent examples and explained it to neuroscience audiences, and how the penalty terms are calculated for AIC and BIC, that would also be interesting.

4. The discussion on AIC and BIC is important was needs more details. Some suggestions to consider that would improve the discussion:

a. Aho K, Derryberry D, Peterson T. Model selection for ecologists: the worldviews of AIC and BIC. Ecology. 2014 Mar 1;95(3):631-6. Discusses the viewpoints of AIC and BIC and how they differ, the various issues etc and what contexts they actually apply in.

b. The phrase from box where “All Models are Wrong and Some are more useful than others” is actually an AIC worldview. In contrast, the common Bayesian (BIC) assumption is that somehow you have the right model in the mixture and all you need to do is find the right model. This distinction would be an interesting discussion point of AIC vs BIC. The issues between consistency and efficiency are also worth noting (these were discussed in the Chandrasekaran et al 2018 paper above)

c. Murtaugh PA. In defense of P values. Ecology. 2014 Mar;95(3):611-7. Derives a detailed analysis of how P values are intimately linked to confidence intervals and to differences in Akaike's information criterion (ΔAIC), two metrics that have been advocated as replacements for the P value.

d. AIC: In Fig. 1a, the authors simulated a very simple tuning example and showed that AIC actually works as intended. So what is the problem here and where is the minefield ? Perhaps actually simulating models where AIC selects the wrong model might be more help to neuroscientists. There are various platitudes about not using AIC in the context of small sample sizes etc, which I think is also true for most basic statistics. A discussion on the relationship between AIC and p-values would also add to this.

e. BIC: There is discussion about BIC, but it would be much more helpful to show a setting where AIC would pick one model and BIC the other model would actually be useful for neuroscientists.

f. There are some consequences of the AIC assumptions: The best model among the ones tested doesn't actually mean that it is a good model. There should be some independent verification of this. The paper could be improved considerably by actually providing examples that test the limits of the proposed model selection approaches.

5. The discussion of clustering and whether nonlinear or linear approaches are important is important in model selection. Some other recent work that talks about this:

a. Lee EK, Balasubramanian H, Tsolias A, Anakwe SU, Medalla M, Shenoy KV, Chandrasekaran C. Non-linear dimensionality reduction on extracellular waveforms reveals cell type diversity in premotor cortex. Elife. 2021 Aug 6;10:e67490. Showed that UMAP on waveforms recorded in cortex performs better than Gaussian Mixture Models on the features and showed how UMAP + Louvain clustering avoids some of the problems of clustering in an embedded space.

b. Sedaghat-Nejad E, Fakharian MA, Pi J, Hage P, Kojima Y, Soetedjo R, Ohmae S, Medina JF, Shadmehr R. P-sort: an open-source software for cerebellar neurophysiology. Journal of neurophysiology. 2021 Oct 1;126(4):1055-75. Showed that UMAP on waveforms recorded in cerebellum is so useful to separate simple and complex spikes.

6. We felt this work could be improved by adding some newer concepts that are especially relevant, and possibly less familiar, to neuroscientists. Tools like DIC, WAIC, and Leave One Out Information Criterion are especially important for latent variable models and doubly stochastic processes where extensive amounts of data analysis is being conducted. Almost all models of dynamics in relevant brain areas assume a latent variable and an emissions process. In these cases, one needs to use techniques such as DIC etc. The primer on these techniques would be important for readers and neuroscientists interested in interpreting data using these techniques.

7. There is a lot of discussion on generalized AICs and sidebar conversation on symmetrizing KL divergence etc and it is unclear how that is relevant to the neuroscience practitioner. The paper alludes to the M-open case but doesn't really explain with an example. Either these are extraneous to the discussion and should be left out, or should be further developed and demonstrated.

8. Minor issues:

Given the discussion on clustering, perhaps it could be added to the title?

Lines 204/205: Please provide some more detailed guidelines on how to answer this question.

Line 43: delete “ref.”

Lines 62/63: Rephrase such that mention name before “he”.

References: Find a way to distinguish the two papers by Kilgard and Merzenich, 1998. Maybe use 1998a and 1998b. Avoid initials (consistent citation style).

Lines 153/4: Better clarify whether BIC refers to the Bayesian Information Criterion (as I understand it) or the Schwarz-Bayesian Information Criterion (usually abbreviated differently).

Line 228: “when” --> “and” (or rephrase sentence, not very clear)

Line 231: “to predicting” --> maybe “via predicting” (from ... via ... to)

Line 273: avoid “and so on (Fig. 2)”. Find better placement of Figure reference.

Line 299: “on the rise” sounds a bit sloppy.

Table 1:

AIC, BIC: better write full names as well

No line below “K-means clustering”

“misleading” --> “suboptimal”

Figure Legend 1b: Right and left are switched.

Figure Legend 2e: Maybe better place webpage (data source) in the main text.

Figure 2: Better combine subplots a and b (both deal with spike sorting).

why do we need to know Akaike's first name or the fact that he developed models to understand rotary kilns etc. This feels like empty text

## Author Response

Rebuttal letter: Navigating the statistical minefield of model selection and clustering in neuroscience

Structure:

Reviewer comments: black, italic

Our replies: blue

Additions to the manuscript text: green

We thank the Editors and the Reviewers for their constructive comments, based on which we believe we could significantly improve our review. All additions to the text are red-lined in the manuscript. Our point-by-point responses are laid out in details below.

Point-by-point response

1. The article currently suffers somewhat from an inconsistent target audience. We felt it lies somewhere between a primer for new students and a state-of-the-art description for experts. It also is not clear whether this is directed towards neuroscientists learning more about the models, or statisticians learning more about neuroscience. To start, we suggest assuming a target audience of neuroscientists, which is the readership of eNeuro. Then improve that particular focus. Currently, most concerns raised are what you would find in any regular stats textbooks, e.g. small sample sizes. This is predictable and does not push the field forward. On the other hand, we felt the introductions and long lists of citations and examples were distracting. Rather than an exhaustive review of examples with many details, this work should focus on the “statistical minefields” and how to avoid them. i.e. more of a statistical primer for neuroscientists, than a neuroscience review for statisticians. Doing so will improve the focus and allow better rigor.

We thank the Editors and Reviewers for this comment. We improved the focus of the article both by complying with the specific points below and by general editing throughout the text. We added specific ‘mines’ including examples where the “true” model is not among the tested ones (Fig. 1b) or where different information criteria favor different models (Fig. 1c, 1e). To raise awareness to these and other pitfalls, we marked and numbered these “mines” in the text and constructed a summary table (new Table 1) of the potential practical problems we discussed. We reduced the number of neuroscience examples; however, as previously we received feedback that pushed us towards including more neuroscience examples, our main goal now was trying to find a good balance.

2. The paper currently does not demonstrate or solve any “minefields” in model selection. All the simulated examples seem to be straightforward and there is no issue there in terms of selecting the right model. This is in stark contrast to recent papers that show specific issues with model selection and cross validation.

The title lures the reader into thinking there are minefields with problems if the wrong model is chosen, but instead the paper itself seems to show that the chosen models always work, which was confusing to the reviewers. With the current title, we would expect some concrete examples of problems and ways to mitigate them. Here are three examples that do so:

a. Chandrasekaran C, Soldado-Magraner J, Peixoto D, Newsome WT, Shenoy KV, Sahani M. Brittleness in model selection analysis of single neuron firing rates. BioRxiv. 2018 Jan 1:430710. Demonstrates that model selection outcomes are dependent on the precise mathematical details of the formulation and if data deviate from the models tested then you get into all sorts of trouble. In particular, the figure 8 in that paper describes the various issues that can happen with model selection.

b. Genkin M, Engel TA. Moving beyond generalization to accurate interpretation of flexible models. Nature Machine Intelligence. 2020 Nov;2(11):674-83. Suggests a data splitting approach and looking for consistency across datasets is the way to find interpretable models.

c. Chari et al., The Specious Art of Single Cell Genomics. Shows how dimensionality reduction applied willy nilly to a 2D space will lead to distortions of the original space, which is essentially a problem of model selection of restricting data to 2 dimensions.

We thank the Reviewer’s for these suggestions. We now discuss these and other pitfalls (e.g. (Evans, 2019; Gronau and Wagenmakers, 2019)), and explicitly draw attention to these issues by marking them as ‘mines’. We constructed a list of 9 common ‘mines’ of model selection in neuroscience with suggestions and examples (Table 1, see below) and explain them in detail in the main text. While we acknowledge the need of revealing the complexity that had probably been too much veiled in the original version, we also intended to keep some of the simpler aspects that we believe make a good introduction into model selection to those neuroscientists who find these concepts less familiar. We hope the Reviewers and Editors will find this compromise acceptable.

Issue Suggestion Example

Mine #1

Selecting models without noticing it

Be aware of the assumptions behind analysis methods. Treat the choice among different algorithms as a model selection problem. (Chari et al., 2021)

Mine #2

Overfitting with overly complex models

Use statistical model selection tools which penalize too many parameters.

polynomial fitting

Mine #3

Selecting from a pool of poorly fitting models might lead to false confidence

Simulate data from each of the tested models multiple times and test whether the real data is sufficient to distinguish across the competing models.

Figure 1b

Mine #4

Different information criteria might favor different models

Consider the strengths and limitations of the different approaches (see Table 2.).

Simulated data can be used to test which model selection method is the most reliable for the given problem.

Figures 1c (AIC favor overfitting), 1e (BIC choose an oversimplified model) and 1f; (Evans, 2019)

Mine #5

Model selection might be sensitive to parameters ignored by the tested models.

Avoid model classes that are too restrictive to account for data heterogeneity. (Chandrasekaran et al., 2018)

Mine #6

Cross-validation techniques are prone to overfitting

A data splitting approach was proposed by Genkin and Engel in which optimal model complexity is determined by calculating KL divergence. (Genkin and Engel, 2020)

Mine #7

Agglomerative hierarchical clustering is sensitive to outliers

Consider divisive methods.

Figure 2c; (Varshavsky et al., 2008)

Mine #8

K-means clustering might converge to local minima

Repeat several times from different starting centroid locations.

Figure 2e, right

Mine #9

Number of clusters not known

Use the elbow method, gap statistics, or model selection approaches.

Figure 2e, left

Table 1. List of common ‘mines’ of model selection and clustering discussed in the paper.

3. Related to #2, perhaps you could provide an example where the true model is not in the classes that were tested and show that model selection provides different answers based on AIC or BIC. We do not demand new experiments/simulations for the resubmission, but include such an example (even one from other publications) would make this point much more rigorously. Alternatively, if you had unpacked the KL divergence with excellent examples and explained it to neuroscience audiences, and how the penalty terms are calculated for AIC and BIC, that would also be interesting.

We now discussed potentially diverging conclusions based on AIC and BIC. We have added new simulations (Fig. 1b, 1c, 1e, 1f; see point #4) and highlighted a thorough treatise of this issue by Nathan Evans (Evans, 2019).

We expanded on the explanation of KL divergence and cross-entropy, which are at the core of AIC derivations. Nevertheless, we are unaware of a clear intuition of ‘why’ the bias correction procedure results in a penalty term that is linear in the number of parameter space dimensions. Our technical understanding is that the bias is (approximately) determined by a quadratic term that is reduced to an identity matrix in the optimal case, yielding an expected value that is its trace, that is, the number of parameters (k). In contrast, while deriving BIC, the likelihood function inherits an O(k) multiplier from the linear term of the Taylor expansion of the log-likelihood, which results in the ln(k) penalty term after taking logarithm. However, this probably does not convey an enlightening insight that merits the detour into more mathematical details.

We added the following paragraph to the main text:

The AIC stands on solid statistical basis, rooted in the Kullback-Leibler divergence (KL) of information theory (Seghouane and Amari, 2007; Seghouane, 2010). The KL divergence quantifies the difference of the true distribution of the data compared to that derived from the tested model. In other words, it can be thought of as the ‘information loss’ (in bits) or the ‘coding penalty’ associated with the imperfect approximation of the true distribution. If we can measure an unbiased empirical distribution, like the frequency of heads and tails when tossing a coin, in the limit of infinite coin flipping, its KL divergence from the true distribution - 0.5 probability for each outcome for a fair coin - will tend to zero (Cavanaugh and Neath, 2019; Shlens, 2014). Formally, the KL divergence of the distribution P from the distribution (model) Q is defined by DKL(P||Q) = ∫ p(x) log p(x) q(x) dx ∞ −∞ Important to the derivation of AIC, the KL divergence can be decomposed to entropy (information content) of P, denoted by H(P) and cross-entropy of P and Q, denoted by H(P,Q): DKL(P||Q) = ∫ p(x) log p(x) dx ∞ −∞ − ∫ p(x) log q(x) dx = H(P,Q) − H(P) ∞ −∞ The model that minimizes this quantity (minimal AIC estimator, MAICE) is accepted (Akaike, 1978, 1974).

Thus, AIC relies on comparing competing models by the difference of their KL divergencies with respect to the ‘true model’ (here denoted by P). It is easy to see that this optimization problem only depends on the cross-entropy term, as the entropy of P is cancelled in the difference. The cross-entropy is mathematically tractable and relatively easy to estimate (Rao and Nayak, 1985); however, the commonly used maximum likelihood estimation is not unbiased in this case, hence it has to be corrected by subtracting the estimated bias. The core of deriving AIC is the bias estimation procedure by approximating the cross-entropy with its Taylor series to the quadratic term, relying also on the central limit theorem and the strong law of large numbers (Akaike, 1973; Cavanaugh, 1997; Konishi and Kitagawa, 2008). This results in the formal definition of AIC as follows:

AIC; = −2 ln(L̂) + 2k where L̂ is the maximum likelihood of the model and k is the number of free parameters (Akaike, 1978, 1974, 1973).

4. The discussion on AIC and BIC is important was needs more details. Some suggestions to consider that would improve the discussion:

a. Aho K, Derryberry D, Peterson T. Model selection for ecologists: the worldviews of AIC and BIC. Ecology. 2014 Mar 1;95(3):631-6. Discusses the viewpoints of AIC and BIC and how they differ, the various issues etc and what contexts they actually apply in.

b. The phrase from box where “All Models are Wrong and Some are more useful than others” is actually an AIC worldview. In contrast, the common Bayesian (BIC) assumption is that somehow you have the right model in the mixture and all you need to do is find the right model. This distinction would be an interesting discussion point of AIC vs BIC. The issues between consistency and efficiency are also worth noting (these were discussed in the Chandrasekaran et al 2018 paper above)

c. Murtaugh PA. In defense of P values. Ecology. 2014 Mar;95(3):611-7. Derives a detailed analysis of how P values are intimately linked to confidence intervals and to differences in Akaike’s information criterion (ΔAIC), two metrics that have been advocated as replacements for the P value.

d. AIC: In Fig. 1a, the authors simulated a very simple tuning example and showed that AIC actually works as intended. So what is the problem here and where is the minefield? Perhaps actually simulating models where AIC selects the wrong model might be more help to neuroscientists. There are various platitudes about not using AIC in the context of small sample sizes etc, which I think is also true for most basic statistics. A discussion on the relationship between AIC and p-values would also add to this.

e. BIC: There is discussion about BIC, but it would be much more helpful to show a setting where AIC would pick one model and BIC the other model would actually be useful for neuroscientists.

f. There are some consequences of the AIC assumptions: The best model among the ones tested doesn’t actually mean that it is a good model. There should be some independent verification of this. The paper could be improved considerably by actually providing examples that test the limits of the proposed model selection approaches.

We thank the Reviewers for these great suggestions. We now discuss AIC and BIC in a more direct comparison, highlighting the ’world view’ interpretation by Aho et al. We discuss situations where AIC and BIC yield different results, aided by new simulations (see point #3 and below). While we very much agree with the statements of Murthaugh’s ’In the defense of P values’ paper, we decided to refrain from discussing the mathematical link between P values and AIC, as we are worried that it would tap into to the (in our opinion largely misguided) ’polemy’ of ’frequentist vs. Bayesian’ statistics, and thus would distract from our main message.

We added the following paragraphs to the main text:

AIC has a penalty of 2k (where k is number of parameters), whereas BIC has a term ln(n)k (where n is the sample size) - thus BIC implies a stronger penalty and hence tends to select simpler models (Brewer et al., 2016) (Fig. 1c-e). Relatedly, AIC is often pictured as a method for selecting models for good ’predictive accuracy’ by penalizing sensitivity to spurious features of the data also known as overfitting, whereas BIC attempts to provide a good description of the fitted data as it panelizes for model complexity more broadly (Evans, 2019; Kass and Raftery, 1995) (but see (Rouder and Morey, 2019)). Mathematically, AIC is ’asymptotically efficient’, minimizing prediction error as sample size tends to infinity, while BIC is ’asymptotically consistent’, selecting the correct model, if it is in the tested pool, as sample size increases.

They can also be seen as representing two different world views: in a complex world where the true generating model is unknown and may be unknowable, as suggested by the quote by Box we cited, one must resort to efficiency, where AIC wins. When one believes that a relatively simple model, included in the set of tested candidates, generates the data, BIC can pick the true model, while AIC does not come with such guarantees (Aho et al., 2014; Chandrasekaran et al., 2018).

Indeed, it is possible that different information criteria will favor different models, without a clear argument on which particular criterion suits the statistical problem at hand best (mine #4). For instance, Evans conducted a systematic comparison of a number of information criteria for a specific class of evidence accumulation models of decision making processes and found that while model selection approaches typically agreed when effect sizes were moderate to large, they could diverge in their conclusions for small or non-existent effects (Evans, 2019). He concluded that one should opt for ’predictive accuracy’ approaches like AIC when the primary goal is to avoid overfitting and thus select a model with strong predictive value, whereas BIC performs better if the goal is to provide the best account of the present data (Evans, 2019; Shmueli, 2010). Going one step further, one might adopt a simulation approach to test which model selection approach is the most reliable for the problem at focus, much the same as in the Evens study.

When complex systems, like those that determine the exact firing activity of neurons, are considered, it is unlikely that our models will capture all aspects of the true generating model. However, model selection approaches will always announce a winner, which raises a set of issues (Chandrasekaran et al., 2018).

First, it is conceivable that all of the tested models fall far from the generating process, in which case model selection will yield a misleading conclusion about the data (mine #3). Second, model selection may be sensitive to parameters of the generating model not captured by the tested models. In such cases, model selection will suggest a model that is closer to the data in statistical or information theoretical terms, but not necessarily conceptually (mine #5). This is detailed in an elegant paper revealing model selection pitfalls when arbitrating between ramp-like and step-like changes in firing rates of single cortical neurons (Chandrasekaran et al., 2018). As a suggestion, one should take multiple close looks at the data, and avoid model classes that are too restrictive to account for data heterogeneity (Chandrasekaran et al., 2018; Genkin and Engel, 2020).

To highlight limitations and ‘mines’, we performed additional simulations. (i) A new example of a tuning curve change has been added (Fig. 1b). In this case we simulated data points by combining and additive and a multiplicative gain and fitted again the purely additive and purely multiplicative models. We demonstrate that although both models show poor fit, AIC still chooses a winner, which might lead to false confidence in this model. We suggest testing whether the data is sufficient to distinguish across the competing models by simulating data from each of the tested models multiple times. (ii) The autoregressive-moving-average (ARMA) model fitting example (Fig. 1c) was supplemented with AIC calculation, which revealed that AIC favored a mode complex model. (iii) The example of fitting a mixture of von Mises distributions to determine the number of modes in a circular distribution (Fig 1d) was repeated for more ambiguous phase preference distribution data, where BIC favored a less complex model than the generating one (Fig. 1e). (iv) We also demonstrate through the same example how low sample size and less distinguishable modes affect the performance of both AIC and BIC (Fig. 1f).

5. The discussion of clustering and whether nonlinear or linear approaches are important is important in model selection. Some other recent work that talks about this:

a. Lee EK, Balasubramanian H, Tsolias A, Anakwe SU, Medalla M, Shenoy KV, Chandrasekaran C. Nonlinear dimensionality reduction on extracellular waveforms reveals cell type diversity in premotor cortex. Elife. 2021 Aug 6;10:e67490. Showed that UMAP on waveforms recorded in cortex performs better than Gaussian Mixture Models on the features and showed how UMAP + Louvain clustering avoids some of the problems of clustering in an embedded space.

b. Sedaghat-Nejad E, Fakharian MA, Pi J, Hage P, Kojima Y, Soetedjo R, Ohmae S, Medina JF, Shadmehr R. P-sort: an open-source software for cerebellar neurophysiology. Journal of neurophysiology. 2021 Oct 1;126(4):1055-75. Showed that UMAP on waveforms recorded in cerebellum is so useful to separate simple and complex spikes.

We thank the Reviewers for suggesting these examples, which are now discussed in the manuscript.

The following text was added:

The advantage of using nonlinear dimensionality reduction algorithms like UMAP for solving neuroscience problems is demonstrated by a novel UMAP-based spike sorting approach that can successfully sort cerebellar Purkinje cell recordings, a notoriously hard problem due the high degree of variability of simple and complex Purkinje cell spikes (Sedaghat-Nejad et al., 2021).

In graph-like data structures where data points (‘nodes’) are connected with links (‘edges’), graph theory-based methods can be applied to detect clusters (‘communities’) of the network. In such methods, a ‘modularity’ measure is optimized that compares the link density inside vs. outside the communities (Blondel et al., 2008). Lee and colleagues applied the graph-based Louvain community detection on spike waveforms of the macaque premotor cortex after non-linear UMAP embedding (see above) and demonstrated the usefulness of this approach in revealing functional cell type diversities (Blondel et al., 2008; Lee et al., 2021). Of note, while the Louvain approach was developed to deal with extremely large graphs in a computationally efficient manner, its two-phase algorithm of finding high modularity partitions leaves the question open whether the order of considering the nodes throughout the algorithm can have a substantial effect on the results (Blondel et al., 2008). Nevertheless, Lee et al. showed that their approach resulted in stable clusters and outperformed Gaussian mixture model clustering applied on specific waveform features (Lee et al., 2021).

6. We felt this work could be improved by adding some newer concepts that are especially relevant, and possibly less familiar, to neuroscientists. Tools like DIC, WAIC, and Leave One Out Information Criterion are especially important for latent variable models and doubly stochastic processes where extensive amounts of data analysis is being conducted. Almost all models of dynamics in relevant brain areas assume a latent variable and an emissions process. In these cases, one needs to use techniques such as DIC etc. The primer on these techniques would be important for readers and neuroscientists interested in interpreting data using these techniques.

We thank the Reviewers for these suggestions. We included a discussion of these novel information criteria using a Bayesian goodness-of-fit measure instead of MLE in the manuscript.

The following paragraph was added to the main text:

The Deviance Information Criterion (DIC) is an extension of AIC, penalizing similarly for model parameters, but applying a different goodness-of-fit measure, defined as the likelihood of the data averaged over the entire posterior distribution (Celeux et al., 2006; Evans, 2019; Spiegelhalter et al., 2002). By departing from the MLE-based goodness-of-fit approach, it gained popularity in Bayesian model selection, to deal with cases where maximum likelihood estimation is difficult (Celeux et al., 2006; Evans, 2019; Li et al., 2020). While DIC assumes approximate normality of the posterior distribution (Spiegelhalter et al., 2014), Watanabe proposed a ‘widely applicable’ (or Watanabe-Akaike) information criterion (WAIC), that does not rely on such assumptions (Watanabe, 2013, 2010a, 2010b). Newest in this family, the ‘leave one out’ information criterion (LOOIC) is similar to WAIC (Gelman et al., 2014; Vehtari et al., 2017; Watanabe, 2010b; Yong, 2018), but it has been proposed to yield more robust results in certain cases (Vehtari et al., 2017). Although these measures incorporate Bayesian notions, they can still be interpreted in terms of predictive accuracy, thus being advanced alternatives of AIC (Evans, 2019). Therefore, Watanabe has made an attempt to generalize BIC as well, which resulted in the ‘widely applicable Bayesian’ information criterion (WBIC) that seeks for the true model instead of minimizing predictive loss (Watanabe, 2013).

Neural dynamics is usually best modelled by latent variable models, assuming a set of interacting hidden and observable variables (Churchland et al., 2012; Pei et al., 2021; Sahani, 1999; Vértes and Sahani, 2018), and doubly stochastic processes, where the dynamics is described by a random point process with varying intensity (Cunningham et al., 2008; Latimer et al., 2015). In these cases, one needs to apply the abovementioned information criteria relying on Bayesian goodness-of-fit estimations (Latimer et al., 2015); therefore, we expect that these novel approaches will soon gain popularity in neuronal modelling and complex data analyses.

7. There is a lot of discussion on generalized AICs and sidebar conversation on symmetrizing KL divergence etc and it is unclear how that is relevant to the neuroscience practitioner. The paper alludes to the M-open case but doesn’t really explain with an example. Either these are extraneous to the discussion and should be left out, or should be further developed and demonstrated. We eliminated most of these discussions to avoid distractions from the main messages.

8. Minor issues:

Given the discussion on clustering, perhaps it could be added to the title? We changed the title to ‘Navigating the statistical minefield of model selection and clustering in neuroscience’.

Lines 204/205: Please provide some more detailed guidelines on how to answer this question.

This is detailed in the ‘Limitations and future directions of AIC’ section. We improved this description and added a reference to this part at the point where the question is posed.

Line 43: delete “ref.”

Deleted.

Lines 62/63: Rephrase such that mention name before “he”.

Rephrased.

References: Find a way to distinguish the two papers by Kilgard and Merzenich, 1998. Maybe use 1998a and 1998b. Avoid initials (consistent citation style).

Fixed.

Lines 153/4: Better clarify whether BIC refers to the Bayesian Information Criterion (as I understand it) or the Schwarz-Bayesian Information Criterion (usually abbreviated differently).

They are the same. Some people abbreviate it as SBIC, which might be confusing, so we consistently use the more widespread BIC abbreviation. We hope this new phrasing is clearer:

In 1978, Schwarz introduced the Bayesian Information Criterion (BIC, also called Schwarz-Bayesian Information Criterion) (Schwarz, 1978).

Line 228: “when” --> “and” (or rephrase sentence, not very clear)

We changed this part.

Line 231: “to predicting” --> maybe “via predicting” (from ... via ... to)

Our understanding is that the structure ’from x to y to z’ is preferred when the list is abstract in the sense that there is no ‘physical journey’ or natural order involved. Something like this example:

“Animals can live anywhere, from forests to deserts to deepest pits of the oceans.” https://itectec.com/englishusage/learn-english-why-are-there-two-tos-in-from-to-to/Line 273: avoid “and so on (Fig. 2)”. Find better placement of Figure reference.

Deleted.

Line 299: “on the rise” sounds a bit sloppy.

Deleted.

Table 1:

AIC, BIC: better write full names as well

Full names have been added.

No line below “K-means clustering”

Fixed.

“misleading” --> “suboptimal”

Rephrased.

Figure Legend 1b: Right and left are switched.

Fixed.

Figure Legend 2e: Maybe better place webpage (data source) in the main text.

We added the link to the main text.

Figure 2: Better combine subplots a and b (both deal with spike sorting).

We reference dimensionality reduction and similarity measures separately in the main text; therefore, we decided to keep these panels separate.

why do we need to know Akaike’s first name or the fact that he developed models to understand rotary kilns etc. This feels like empty text

We found it interesting to understand how specific industrial applications, important at one time, could drive statistics as a field forward in a direction that has become, probably beyond the expectations of the statisticians themselves, important to an exceptionally broad range of disciplines. Nevertheless, we removed the statement as it is indeed tangential to our main points.

References

Aho K, Derryberry D, Peterson T (2014) Model selection for ecologists: The worldviews of AIC and BIC. Ecology 95:631-636.

Akaike H (1978) A Bayesian Analysis of the Minimum AIC Procedure. Ann Inst Stat Math 30:9-14.

Akaike H (1974) A new look at the statistical model identification. IEEE Trans Automat Contr 19:716-723.

Akaike H (1973) Information theory and an extension of the maximum likelihood principle In: 2nd International Symposium on Information Theory , pp267-281.

Blondel VD, Guillaume J-L, Lambiotte R, Lefebvre E (2008) Fast unfolding of communities in large networks. J Stat Mech Theory Exp 2008:P10008.

Brewer MJ, Butler A, Cooksley SL (2016) The relative performance of AIC, AIC C and BIC in the presence of unobserved heterogeneity. Methods Ecol Evol 7:679-692.

Cavanaugh JE (1997) Unifying the derivations for the Akaike and corrected Akaike information criteria. Stat Probab Lett 33:201-208.

Cavanaugh JE, Neath AA (2019) The Akaike information criterion: Background, derivation, properties, application, interpretation, and refinements. Wiley Interdiscip Rev Comput Stat 11:1-11.

Celeux G, Forbes F, Robert CP, Titterington DM (2006) Deviance Information Criteria for Missing Data Models. Bayesian Anal 1:651-674.

Chandrasekaran C, Soldado-Magraner J, Peixoto D, Newsome WT, Shenoy K V., Sahani M (2018) Brittleness in model selection analysis of single neuron firing rates. bioRxiv 430710.

Chari T, Banerjee J, Pachter L (2021) The Specious Art of Single-Cell Genomics. BioRxiv 1-25.

Churchland MM, Cunningham JP, Kaufman MT, Foster JD, Nuyujukian P, Ryu SI, Shenoy K V. (2012) Neural population dynamics during reaching. Nature 487:51-56.

Cunningham JP, Shenoy K V., Sahani M (2008) Fast Gaussian process methods for point process intensity estimation In: Proceedings of the 25th International Conference on Machine Learning , pp192-199.

Evans NJ (2019) Assessing the practical differences between model selection methods in inferences about choice response time tasks. Psychon Bull Rev 26:1070-1098.

Gelman A, Hwang J, Vehtari A (2014) Understanding predictive information criteria for Bayesian models. Stat Comput 24:997-1016.

Genkin M, Engel TA (2020) Moving beyond generalization to accurate interpretation of flexible models. Nat Mach Intell 2:674-683.

Gronau QF, Wagenmakers E-J (2019) Limitations of Bayesian Leave-One-Out Cross-Validation for Model Selection. Comput Brain Behav 2:1-11.

Kass RE, Raftery AE (1995) Bayes Factors. J Am Stat Assoc 90:773.

Konishi S, Kitagawa G (2008) Information Criteria and Statistical Modeling, Springer Series in Statistics. New York, NY: Springer New York.

Latimer KW, Yates JL, Meister MLR, Huk AC, Pillow JW (2015) Single-trial spike trains in parietal cortex reveal discrete steps during decision-making. Science (80- ) 349:184-187.

Lee EK, Balasubramanian H, Tsolias A, Anakwe SU, Medalla M, Shenoy K V., Chandrasekaran C (2021) Non-linear dimensionality reduction on extracellular waveforms reveals cell type diversity in premotor cortex. Elife 10.

Li Y, Yu J, Zeng T (2020) Deviance information criterion for latent variable models and misspecified models. J Econom 216:450-493.

Pei F, Ye J, Zoltowski D, Wu A, Chowdhury RH, Sohn H, O’Doherty JE, Shenoy K V., Kaufman MT, Churchland M, Jazayeri M, Miller LE, Pillow J, Park IM, Dyer EL, Pandarinath C (2021) Neural Latents

Benchmark ‘21: Evaluating latent variable models of neural population activity In: 35th Conference on Neural Information Processing Systems (NeurIPS 2021) , pp1-37.

Rao CR, Nayak TK (1985) Cross Entropy, Dissimilarity Measures, and Characterizations of Quadratic Entropy. IEEE Trans Inf Theory 31:589-593.

Rouder JN, Morey RD (2019) Teaching Bayes’ Theorem: Strength of Evidence as Predictive Accuracy. Am Stat 73:186-190.

Sahani M (1999) Latent variable models for neural data analysis.

Sedaghat-Nejad E, Fakharian MA, Pi J, Hage P, Kojima Y, Soetedjo R, Ohmae S, Medina JF, Shadmehr R (2021) P-sort: An open-source software for cerebellar neurophysiology. J Neurophysiol 126:1055- 1075.

Seghouane A-K, Amari S-I (2007) The AIC criterion and symmetrizing the Kullback-Leibler divergence. IEEE Trans Neural Netw 18:97-106.

Seghouane AK (2010) Asymptotic bootstrap corrections of AIC for linear regression models. Signal Processing 90:217-224.

Shlens J (2014) Notes on Kullback-Leibler Divergence and Likelihood. arXiv 1-4.

Shmueli G (2010) To explain or to predict? Stat Sci 25:289-310.

Spiegelhalter DJ, Best NG, Carlin BP, Van der Linde A (2014) The deviance information criterion: 12 years on. J R Stat Soc Ser B Stat Methodol 76:485-493.

Spiegelhalter DJ, Best NG, Carlin BP, Van Der Linde A (2002) Bayesian measures of model complexity and fit. J R Stat Soc Ser B Stat Methodol 64:583-616.

Varshavsky R, Horn D, Linial M (2008) Global Considerations in Hierarchical Clustering Reveal Meaningful Patterns in Data. PLoS One 3:e2247.

Vehtari A, Gelman A, Gabry J (2017) Practical Bayesian model evaluation using leave-one-out cross-validation and WAIC. Stat Comput 27:1413-1432.

Vértes E, Sahani M (2018) Learning Doubly Intractable Latent Variable Models via Score Matching In: Proceedings of 32nd Conference on Neural Information Processing Systems (NeurIPS 2018) , pp1-4.

Watanabe S (2013) A widely applicable bayesian information criterion. J Mach Learn Res 14:867-897.

Watanabe S (2010a) Equations of states in singular statistical estimation. Neural Networks 23:20-34.

Watanabe S (2010b) Asymptotic equivalence of Bayes cross validation and widely applicable information criterion in singular learning theory. J Mach Learn Res 11:3571-3594.

Yong L (2018) LOO and WAIC as Model Selection Methods for Polytomous Items. arXiv 1-39.
